# Psychometric properties of the Spanish SABA Reliance Questionnaire (SRQ) among patients with asthma

**DOI:** 10.1016/j.jacig.2022.10.008

**Published:** 2023-01-20

**Authors:** Mar Martínez, Juan Carlos López, Jesús Molina, Mónica Sorribas, Mario Arancón, Raúl de Simón, David Díaz, Eva Trillo-Calvo, José Tomás Gómez, Francisco Fernández-Conde, Marta Alegría, Maite Artés, Cristina Calle, Holly Foot, Joaquín Sánchez-Covisa

**Affiliations:** a Centro de Salud Zorroza, Bilbao, Spain; b Centro de Salud Cotolino, Castro Urdiales, Cantabria, Spain; c Centro de Salud Francia, Fuenlabrada, Madrid, Spain; d Centro de Salud Alhama de Murcia, Murcia, Spain; e Centro de Salud Tres Cantos, Madrid, Spain; f Centro de Salud Luis Vives, Alcalá de Henares, Madrid, Spain; g Centro de Salud Los Barrios, Algeciras, Spain; h Centro de Salud Campo de Belchite, Zaragoza, Spain; i Centro de Salud Nájera, Nájera, La Rioja, Spain; j AstraZeneca Farmacéutica Spain S.A., Madrid, Spain; k IQVIA RDS Spain SL, Madrid, Spain; l Adelphi Targis, Barcelona, Spain; m Spoonful of Sugar, Hove, United Kingdom; n School of Pharmacy, The University of Auckland, Auckland, New Zealand

**Keywords:** Asthma, short-acting bronchodilator, SABA, overreliance, patient belief, patient attitude, SABA Reliance Questionnaire

## Abstract

**Background:**

Patient beliefs about their asthma and its treatment may contribute to overreliance on short-acting β_2_-agonist (SABA) therapy, leading to increased risk for potentially life-threatening exacerbations. The SABA Reliance Questionnaire (SRQ) is a validated tool for evaluating patients beliefs about SABAs that may lead to overreliance and overuse.

**Objective:**

Our aim was to evaluate the psychometric properties of the Spanish version of the SRQ.

**Methods:**

This was an observational, cross-sectional, single-country questionnaire validation study in adults with asthma. Reliability (ordinal α) and validity (convergent and discriminant) of SRQ were evaluated. Concurrent validity was assessed with the Beliefs about Medication Questionnaire, the Treatment Satisfaction Questionnaire for Medication, and a visual analog scale item to assess patients’ perceptions of the importance of their reliever inhaler. Discriminant validity was assessed through differences in mean SRQ sum score between patients with high adherence to inhaled corticosteroids and those with low adherence, as measured by the Medication Adherence Report Scale-9 and the Test of Adherence to Inhalers.

**Results:**

The Spanish-SRQ exhibited good psychometric properties among 131 patients with asthma. Internal consistency was confirmed with an ordinal α of 0.85. All 5 items were useful for measuring patients’ beliefs about SABAs that may lead them to be overreliant on SABAs. Concurrent validity with the Beliefs about Medication Questionnaire, Treatment Satisfaction Questionnaire for Medication, and a visual analog scale item assessing patients’ perceptions of the importance of their reliever inhaler was demonstrated.

**Conclusion:**

The Spanish version of the SRQ is a valid tool for evaluating potential overreliance on SABAs in Spanish-speaking patients to enable early intervention and support.

Asthma is a chronic inflammatory disease of the airways resulting in bronchial hyperresponsiveness and variable air flow obstruction.^1^ In 2015, almost 0.4 million people globally died as a result of their asthma, a decrease of 26.7% from 1990, even though the prevalence of asthma increased by 12.6% in the same time period.^2^ Symptoms include wheeze, shortness of breath, chest tightness, and/or cough. Patients may also experience episodic exacerbations of asthma that may be life-threatening.^3^ Because asthma is a chronic disease, the objective of asthma management is to achieve and maintain symptom control and prevent or reduce the risk of future potentially life-threatening exacerbations.^3^ Until recently, the standard treatment approach for asthma of all levels of severity included short acting β_2_-agonists (SABAs) as needed as a reliever medication. With the 2021 Global Initiative for Asthma, however, there has now been a dramatic shift.^3^ SABAs are no longer recommended as the preferred reliever for patients who are symptomatic, and they should not be used as monotherapy because of significant safety concerns and poor outcomes. Instead, adults and adolescents with asthma should receive inhaled corticosteroid (ICS)-containing controller treatment to reduce their risk of serious exacerbations and control their symptoms.^3^ An ICS-containing controller can be delivered either with regular daily treatment or, in cases of mild asthma, with ICS-formoterol taken as required for symptom relief. Compared with a SABA alone, low-dose ICS-formoterol as the reliever reduces the risk of severe exacerbations, with similar symptom control and similar lung function.^3^

High levels of SABA use (also referred to as SABA overreliance) is a marker of poor asthma control and an indicator of an increased risk of potentially life-threatening asthma exacerbation.^4^ A recent study among more than 365,000 patients with asthma found that compared with those who used no more than 2 SABA canisters per year, those who used 3 or more SABA canisters per year had an incremental risk of death (with use of 3-5 canisters per year, the hazard risk [HR] was 1.26 [95% CI = 1.14-1.39]; with use of 6-10 canisters per year, HR = 1.67 [95% CI = 1.49-1.87]; and with use of ≥11 canisters per year, HR = 2.35 [95% CI = 2.02-2.72]).^5^ A longitudinal retrospective study in Spanish primary and specialized care settings estimated that the prevalence of asthma in Spain is 5.3% (95% CI = 5.1-5.5) and SABA overreliance is prevalent, with 28.7% (95% CI = 27.7-29.7) of patients using 3 or more SABA canisters per year.^6^ Data from the SABA use IN Asthma (SABINA) studies revealed that SABA overreliance is prevalent across European countries, with an estimated one-third of patients with asthma using 3 or more SABA canisters per year.^7^ Results from the SABA use IN Asthma study also confirmed notable overuse of SABA in asthmatic patients in Spain, with 5.1% of patients prescribed 12 or more cannisters per year. Therefore, the inappropriate use of SABA remains problematic in a significant percentage of patients with asthma, and it is associated with increased use of medical care and a higher risk of adverse outcomes.

SABA overreliance is likely related to patients' beliefs about their asthma and its treatment. Patients’ beliefs have been shown to influence treatment engagement and adherence.^8^ Many patients see asthma as a short-term episodic condition rather than a long-term condition, thus reinforcing reliance on SABA based on personal need for rapid symptom relief.^9^ Also, many patients have a strong emotional attachment to SABA relievers that is driven by the relievers' efficacy and success in quickly alleviating asthma symptoms, allowing patients the freedom to continue with their daily lives.^10^ Therefore, assessing individual patient beliefs can help identify patients at risk of SABA overreliance and allow early intervention. One such validated tool developed for the evaluation of patients' beliefs around reliever medication is the 5-item SABA Reliance Questionnaire (SRQ).^9^ The SRQ specifically assesses patients’ beliefs about SABAs that might lead them to be overly reliant and thus inform health interventions to reduce inappropriate medication use and improve asthma management.^9^ The SRQ has been shown to have good internal reliability (Cronbach α = 0.74) and both criterion and discriminant validity as assessed by positive correlation with the visual analog scale (VAS) item “How important is your reliever (SABA) medication?” (*r* = 0.216; *P* < .0001) and negative correlation with the Medication Adherence Report Scale (MARS)-ICS (adherence) scores (*r* = –0.291; *P* < .0001). These observations indicate that patients with stronger beliefs in the personal necessity of a SABA (those with high SRQ scores) are significantly more likely to self-report low adherence to ICS therapy.

Here we report the results of a study undertaken to evaluate the psychometric properties, reliability, validity, and internal consistency of a Spanish version of the SRQ.

## Methods

This was an observational, cross-sectional, multicenter, single-country study undertaken to determine the psychometric properties and validity of the Spanish version of the SRQ, which was developed to enable evaluation of overreliance on SABA among Spanish-speaking patients with asthma (see Table E1 in the Online Repository at www.jaci-global.org). Before the psychometric validation study, a linguistic and cultural adaptation of the original English version of Reliever Reliance Test (RRT) questionnaire (which includes the SRQ) was performed following the methodology recommended by the International Society for Pharmacoeconomics and Outcomes Research (see Table E2 in the Online Repository at www.jaci-global.org).^11^

The study received ethics approval by Centro de Estudos E Investigação Científica Center for Scientific Studies and Research Aragón [Center for Scientific Studies and Research Aragón] on April 21, 2021.

### Study population

Eligible participants were adults (aged ≥18 years) with a confirmed diagnosis of asthma (regardless of severity stage) who were currently receiving SABA as part of their asthma management and attending a routine asthma-related health care consultation with their primary care physician at one of the participating centers (on-site or remotely). In addition, patients were required to have been prescribed a SABA at least 12 months before inclusion, with at least 12 months' availability of electronic medical records, to be receiving an ICS for maintenance treatment, and to be able to complete the SRQ questionnaire and other patient-reported outcome (PRO) questionnaires using an online survey platform. Patients currently enrolled in a clinical trial, those receiving institutional care, and those with mental illnesses that would prevent them from correctly completing the questionnaires were excluded from participation. All eligible patients provided informed consent before participation.

### Data collection

Data were collected at 9 primary care centres in Spain. At the study visit, patients were asked to complete a set of questionnaires, including the SRQ, the Beliefs about Medication Questionnaire (BMQ), the MARS-9, the Test of Adherence to Inhalers (TAI), the Treatment Satisfaction Questionnaire for Medication (TSQM) and the VAS item “How important is your reliever (SABA) medication?” Because of the COVID pandemic, the study visit was conducted either by telephone or videoconference. All PRO questionnaires were completed online by the patient via a secure link sent to each patient and associated with the patient identifier in order to merge the PRO information with an electronic case report form.

### Analyses

The acceptability (feasibility) of the SRQ was evaluated by estimating the percentage of complete responses and the ceiling and floor effects. The reliability of the SRQ was evaluated by estimating the internal consistency. Concurrent validity was assessed with the BMQ-Specific subscale (referring to SABAs), the TSQM, and a VAS item to assess patients’ perceptions of the importance of their reliever inhaler. Discriminant validity was assessed by comparing SRQ scores depending on patient’s adherence to ICS, as measured by MARS-9 questionnaire and TAI (12 items). The conceptual hypotheses informing these assessments is that patients overrelying on SABA (those with high SRQ scores) will also have high scores on the BMQ-Necessity subscale, the TSQM, and the VAS item, because they have strong beliefs regarding the necessity of SABA medication (as measured by the BMQ-Necessity subscale) and are satisfied with it (as measured by the TSQM). Also, it was hypothesized that patients overrelying on their SABA would be more likely to have poor adherence to maintenance ICS therapy, resulting in a negative association between patients scoring high on the SRQ and adherence to ICS therapy, as measured by MARS-9 and TAI. All analyses were based on the full analysis set (all patients).

### Statistical analysis

All variables were analyzed descriptively with appropriate statistical methods: categoric variables were analyzed by frequency tables (absolute and relative frequencies) and continuous variables were analyzed by sample statistics (ie, mean, SD, minimum, median, quartiles, and maximum).

Several hypothesis tests were set *a priori* to evaluate the convergent and discriminant validity of the Spanish version of SRQ. These included testing of the null hypothesis for concurrent validity and the null hypothesis for discriminant validity. The null hypothesis for concurrent validity was defined as a Pearson correlation between the SRQ total score and the BMQ-Specific VAS item to assess patients’ perceptions of the importance of their reliever inhaler and a TSQM score of 0 (indicating no concurrent validity). The null hypothesis for discriminant validity was defined as the difference between means (or medians in cases in which the distribution is not normal) for the SRQ total score in patients with good adherence to ICSs (higher scores on the MARS-9 and/or TAI) versus patients with poor adherence (low scores on the MARS-9 and/or TAI) of 0 (indicating no discriminant validity). The cutoff scores for low and high adherence were determined by the sample responses to the MARS-9 by calculating the maximum potential score on the MARS-9^9^^,^^12^ and identifying those participants scoring within the highest third (considered to have high adherence to ICS therapy) and the lowest third (considered to have low adherence to ICS therapy), respectively. The cutoffs for the TAI for high and low adherence were 50 and 45 or lower, respectively.^13^ In addition, hypothesis testing was conducted to explore potential factors influencing the SRQ total score, with a null hypothesis that all potential factors had a β-coefficient equal to 0 in the multiple regression model. The significance level for all hypothesis testing was set at .05 (2-tailed testing).

The feasibility of the questionnaire was assessed by calculating the percentage of patients not answering all items of the SRQ. Floor and ceiling effects were assessed by calculating the percentage of patients answering the lowest-option response in all the items of the SRQ and the highest-option response, respectively and their 95% CI. Internal consistency was assessed using ordinal α for the whole SRQ questionnaire. Also, ordinal α excluding every item was calculated to evaluate the consistency of the item with the scale. Concurrent validity was evaluated by calculating the Pearson or Spearman correlation (depending on the variable distribution) of the overall score of SRQ with the validated Spanish versions of the BMQ-Specific, the VAS item to assess patients’ perceptions of the importance of their reliever inhaler, and the TSQM. Discriminant validity was evaluated by using the Student *t* test to compare the overall mean score of SRQ in patients with good adherence to ICS therapy (as measured by the score in the MARS-9 adherence questionnaire in percentile 66 [the highest third]) versus in patients showing no good adherence (percentile 33 of the score in MARS-9 [the lowest third]). The Student *t* test was also used to compare the overall mean score of SRQ in patients with good adherence to ICS (as measured by a score of 50 on the TAI) versus in patients showing no good adherence (a score of ≤45 on the TAI). To further understand the relationship between SRQ scores and adherence, an exploratory ANOVA or Kruskal-Wallis test (in cases in which assumptions for parametric analyses were not fulfilled) was conducted. In this way, the mean SRQ scores among the different types of patient nonadherence (“erratic noncompliance,” defined as a score of <25 on items 1-5; “on-purpose noncompliance,” defined as a score of <25 on items 5-10; and “unconscious noncompliance,” defined as a sore of <4 on items 11 and 12), as defined by the TAI, were compared. To check whether using different definitions of high and low adherence would have an impact on the analysis, a sensitivity analysis was conducted by using different MARS-9-ICS cutoff points; high and low adherence groups were defined as those scoring in the top and bottom 30% of the sample (as opposed to in the top and bottom thirds).

### Sample size and power calculations

No definite guidelines exist regarding required sample size for psychometric validation. However, the sample size for the psychometric validation of a questionnaire should be large enough to meet a desired level of measurement precision or SE. Sample sizes of around 200 participants for psychometric analyses have been proposed, although the sample size requirements for reliability and validity assessment depend on the specific circumstances and analytical tools. For the current study, because the SRQ scale consists of only 5 items, a factor analysis was not considered necessary and a smaller sample size would be acceptable. Most of the psychometric indicators for reliability and validity in our study were based on correlation coefficients, and a sample size of 124 patients was anticipated to provide 80% power (α = 0.05; 2-tailed test) to detect a correlation between 2 variables of 0.25 as being statistically significant. A sample size of 124 patients was also anticipated to be sufficient for the exploratory objective to assess the mean difference between the SRQ total score of patients with good adherence to ICS and nonadherent patients, as this sample would be sufficient to detect a difference of 0.50 SD (effect size) between 2 independent groups of subjects of equal size with an α risk of 0.05 and a statistical power of 0.80.

## Results

**Fig 1:**
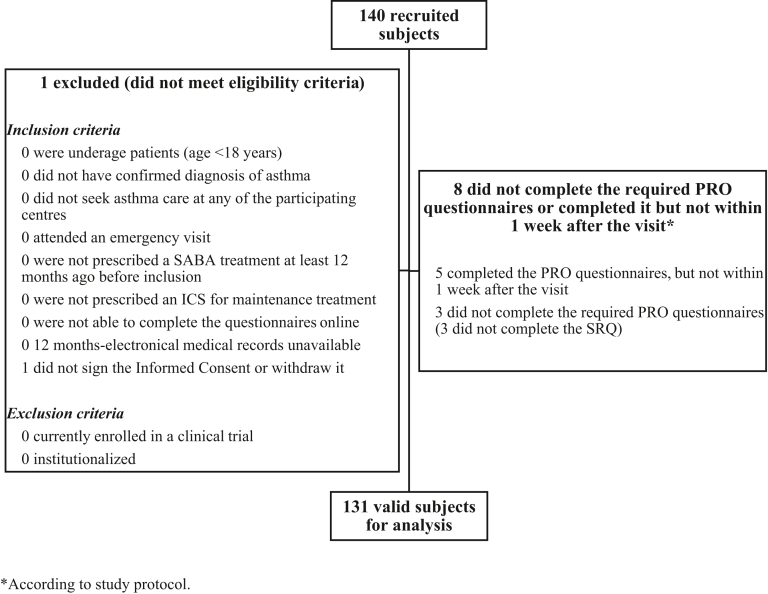
Valid sample for analysis.

A total of 131 subjects with a confirmed diagnosis of asthma (regardless of the severity stage), with SABA prescriptions, and seeking asthma care at any of the participant sites were recruited in the study and included in the analyses (Fig 1). The amount of missing data was low, with only 1 patient not completing either the TSQM or the MARS-9. Consequently, imputation using the population’s mean score for the participant missing data was not considered necessary.

### Demographics, anthropometric characteristics, and clinical history

The characteristics of the participating patients are described in Table I. The mean (± SD) time from diagnosis of asthma was 19.8 (12.3) years. Most patients presented with mild (n = 56 [42.7%]) or moderate (n = 66 [50.4%]) asthma and were currently using a mean (± SD) of 3 types of asthma treatments, mainly long-acting β-agonists (n=112 [85.5%]) in addition to ICSs and SABAs. Comorbidities were present in 31.3% of patients. The most common comorbidities were nose and sinus disorders (26.8%), obesity (39%), and anxiety (34.2%).

**Table I:** Characteristics and clinical history of the study population

Characteristic	Study population (N = 131)
Sex, no. (%)	
Male	46 (35.1)
Female	85 (64.9)
Age (y)	
Mean (SD)	45.6 (14.7)
Median (min, max)	46 (18, 84)
Education level, n (%)	
Without studies (did not complete primary education)	2 (1.5)
Primary education	32 (24.4)
Secondary education	13 (9.9)
Higher secondary education	45 (34.4)
University education	39 (29.8)
BMI (kg/m^2^)	
Mean (SD)	27.2 (5.1)
Median (min, max)	26.8 (19.4, 40.9)
Smoking status, no. (%)	
Never	74 (56.5)
Current	25 (19.1)
Former	32 (24.4)
Smoking history among current or former smokers	
Length of smoking history (y)	
Mean (SD)	19.4 (12.7)
Median (min, max)	20 (2, 50)
Cigarettes/d	
Mean (SD)	13.5 (10.3)
Median (min, max)	12 (2, 60)
Time since diagnosis of asthma (y)	
Mean (SD)	19.8 (12.3)
Median (min, max)	17 (1.2, 63.7)
Asthma severity, no. (%)	
Mild	56 (42.7)
Moderate	66 (50.4)
Severe	9 (6.9)
Previous asthma treatments	
Mean no. (SD)	2.98 (1.14)
Median no. (min, max)	3 (1, 6)
Type of treatment, no. (%)	
ICS	113 (86.3)
SCS	30 (22.9)
SABA	125 (95.4)
LABA	93 (71.0)
LRA	25 (19.1)
Inhaled short-acting anticholinergic	4 (3.1)
mAb	1 (0.8)
Current asthma treatment	
Mean no. (SD)	3.1 (0.59)
Median no. (min, max)	3 (2, 6)
Type of treatment, no. (%)	
ICS	131 (100)
SCS	5 (3.8)
SABA	131 (100)
LABA	112 (85.5)
LRA	17 (13.0)
Inhaled short-acting anticholinergic	2 (1.5)
mAb	1 (0.8)
Hospitalizations during the previous 12 mo	
Mean no. (SD)	0.14 (1.32)
Median no. (min, max)	0 (0, 15)
Exacerbations during the previous 12 mo∗	
Mean no. (SD)	0.73 (1.76)
Median no. (min, max)	0 (0, 15)
Comorbidities	
≥1 comorbidity, no. (%)	41 (31.3)
Mean no. (SD)	0.56 (1.01)
Median no. (min, max)	0 (0, 5)
Type, no. (%)	
Nose and sinus disorder	11 (26.8)
Gastroesophageal reflux	5 (12.2)
Obesity	16 (39.0)
Sleep apnoea syndrome	5 (12.2)
Anxiety	14 (34.2)
Depression	2 (4.9)
Fibromyalgia	1 (2.4)
Other	19 (46.3)

*BMI*, Body mass index; *LABA*, long-acting β-agonist; *LRA*, leukotriene receptor antagonist; *max*, maximum; *min*, minimum; SCS, systemic corticosteroid.

∗ Severe exacerbations were defined as those requiring the use of an SCS (oral, suspension, or injection) or a dose increase of maintenance therapy for at least 3 days, or hospitalization or emergency room visits due to asthma requiring the use of and SCS. Moderate and mild exacerbations were defined as events requiring additional treatment (oral corticosteroids) to prevent progression to severe exacerbation.

### Intensity of asthma symptoms and SABA use during the week before SRQ evaluation

In general, patients did not present with asthma symptoms within the week before the study visit. Among those experiencing asthma symptoms, the intensity was generally low or moderate (Table II). SABA use and asthma characteristics are described in Table II. Most patients did not use a SABA within the week before the study visit (52.7%) or used it 2 or fewer times (25.2%). The mean number of days with daytime asthma symptoms, mean number of asthma attacks, and mean number of times patients reported being woken during the night because of their asthma symptoms were low (Table II).

**Table II:** Intensity of asthma symptoms in the previous week, SABA use, and asthma characteristics

Symptom and intensity level in previous week	Study population (N = 131)
Chest tightness or pressure, no. (%)	
None	80 (61.1)
Mild	42 (32.1)
Moderate	7 (5.3)
Severe	2 (1.5)
Difficulty breathing, no. (%)	
None	69 (52.7)
Mild	48 (36.6)
Moderate	13 (9.9)
Severe	1 (0.8)
Wheezing, no. (%)	
None	75 (57.3)
Mild	51 (38.9)
Moderate	4 (3.1)
Severe	1 (0.8)
Shortness of breath after exertion, no. (%)	
None	58 (44.3)
Mild	57 (43.5)
Moderate	14 (10.7)
Severe	2 (1.5)
Dry cough, no. (%)	
None	73 (55.7)
Mild	46 (35.1)
Moderate	10 (7.6)
Severe	2 (1.5)
SABA use, no. (%)	
None	69 (52.7)
≤2	33 (25.2)
3	14 (10.7)
4 or 5	5 (3.8)
>5	10 (7.6)
Days with asthma symptoms	
Mean (SD)	1.6 (2.06)
Median (min, max)	1 (0, 7)
No. of asthma attacks	
Mean (SD)	0.52 (1.36)
Median (min, max)	0 (0, 7)
Nighttime awakenings due to asthma symptoms	
Mean (SD)	0.37 (1.11)
Median (min, max)	0 (0, 7)

*max*, Maximum; *min*, minimum.

### Psychometric analysis of the SRQ

All patients answered all questions on the SRQ, indicating a good level of understanding and acceptable feasibility. The distribution of SRQ scores is shown in Table E3 and Fig E1 (both of which are available in the Online Repository at www.jaci-global.org). Floor and ceiling effects are shown in Table III. The proportions of patients scoring the lowest-option response in all the items of the SRQ (floor effect) and the highest-option response (ceiling effect) were low, at 3.82% of patients and 1.53%, respectively (Table III).

**Table III:** Description and floor and ceiling effect of the SRQ

Item	Mean (SD)	Median (min, max)	95% CI	Floor effect (%)∗ (N = 131)	Ceiling effect (%)† (N = 131)
1	3.37 (1.30)	4 (1, 5)	3.14-3.59	11.45	22.14
2	3.34 (1.29)	4 (1, 5)	3.12-3.57	11.45	19.85
3	2.85 (1.32)	3 (1, 5)	2.62-3.07	20.61	12.21
4	3.21 (1.11)	3 (1, 5)	3.01-3.40	8.40	8.40
5	2.91 (1.41)	3 (1, 5)	2.66-3.15	22.14	15.27
Total score	15.67 (4.94)	5 (17, 25)	14.82-16.52	3.82	1.53

∗ Proportion of patients with an SRQ score of 5.

† Proportion of patients with an SRQ score of 25.

Analysis of the internal reliability of the SRQ is shown in Table IV. The ordinal α was 0.85, indicating good internal consistency. All 5 items were useful for measuring patients’ beliefs about SABAs that might lead them to overreliance.

**Table IV:** Internal reliability of the SRQ

Internal reliability of the SRQ, if	SRQ total score, mean	SRQ total score, SD	Ordinal α (*P* value)
	—	—	.85
Item 1 is deleted	12.13	3.95	.79
Item 2 is deleted	12.33	4.04	.82
Item 3 is deleted	12.82	4.00	.81
Item 4 is deleted	12.47	4.20	.83
Item 5 is deleted	12.76	3.99	.83

The SRQ total score demonstrated concurrent validity with the effectiveness of the BMQ-Necessity subscale *(r* = 0.4481; *P* < .0001), the VAS item (*r* =2 312; *P* = .008), and the TSQM (*r* = 2353; *P* = .007) and global satisfaction (*r* = 2657; *P* = .002) dimensions (Table V).

**Table V:** Correlation between SRQ score and other PRO questionnaires assessing patients’ perceptions of their medications

PRO questionnaire	Correlation with SRQ	
	*r*	(*P* value)∗
VAS item	0.2312	.008
BMQ-specific		
Specific necessity scale	0.4481	<.0001
TSQM		
Effectiveness	0.2353	.007
Side effects	0.1360	.123
Convenience	0.1433	.104
Global satisfaction	0.2657	.002

∗ Spearman correlation.

No statistically significant differences were observed for the overall mean score of SRQ among patients with good adherence to ICS therapy (as measured by score on the MARS-9 adherence questionnaire in percentile 66 [the highest third] or by a score of 50 on the TAI) versus among patients showing no good adherence (percentile 33 of the score in MARS-9 [the lowest third] or a score of 45 or lower on the TAI [Table VI]). All of the patients met the criterion of the erratic noncompliance pattern, and all of them also met the criterion of the on-purpose noncompliance pattern. Thus, the descriptive analysis of both groups are the descriptive data for the total sample. Almost all of the patients (n = 130) met the criterion for an unconscious noncompliance pattern.

**Table VI:** Discriminant validity comparison of the overall mean SRQ score between patients with low versus high adherence to ICS therapy according to the MARS-9 score and the TAI and among different types of noncompliance patterns according to the TAI

Patient subgroup	n	SRQ score		*P* value∗
		Mean (SD)	Median (min, max)	
According to MARS-9 score				.3426†
≤ Percentile 33 (low adherence to ICS therapy)	43	16.35 (4.13)	17 (6, 25)	
≥ Percentile 66 (high adherence to ICS therapy)	50	15.42 (5.11)	16 (5, 24)	
According to the TAI				.3588†
≤45 (low adherence to ICS)	72	16.28 (4.49)	17 (5, 25)	
50 (high adherence to ICS)	21	15.19 (5.59)	17 (5, 23)	
Noncompliance pattern (TAI)‡				.2200#
Erratic§	131	15.67 (4.94)	17 (5, 25)	
On purpose‖	131	15.67 (4.94)	17 (5, 25)	
Unconscious¶	130	15.63 (4.93)	17 (5, 25)	

∗ *P* value compares the mean (calculated using a nonparametric Wilcoxon-Mann-Whitney test) between 2 groups because the group with an erratic noncompliance pattern and the group with an on-purpose noncompliance pattern are equal.

† According to the Student *t* test.

‡ A patient may have more than 1 noncompliance pattern.

§ A score of 5 to 25 on items 1 to 5.

‖ A score of 5 to 25 on items 6 to 10.

¶ A score of 2 to 4 on items 11 and 12.

# According to the Wilcoxon-Mann-Whitney test.

## Discussion

The psychometric evaluation reported here showed that the Spanish version of the SRQ exhibits good psychometric properties in terms of acceptability, reliability, and concurrent validity with the BMQ-Specific subscale (referring to SABAs), the TSQM effectiveness and global satisfaction dimensions, and a VAS item assessing patients’ perceptions of the importance of their reliever inhaler. These results support the use of the Spanish version of the SRQ for evaluation of Spanish-speaking patients for their potential overreliance on SABA as part of their asthma self-management. Identification of patients at risk for SABA overreliance can enable early interventions, including education and support to encourage patients to adhere to preventer medication regimens for symptom control consistent with current treatment guidelines.^3^ The SRQ forms part of the RRT, a patient-friendly tool, consisting of the SRQ, a single question assessing SABA use, and contextual information to help patients understand what their personal questionnaire responses might mean for them.^14^ Together, these components form a short test designed to assess what patients think of their SABA inhaler and whether they may be relying on it too much to relieve their asthma symptoms. It includes recommendations to patients indicating when they should seek help from a health care professional, supporting its potential utility as a pragmatic and valuable tool in primary care for identifying patients' beliefs that may put them at risk of SABA overreliance and to flag those who would benefit from an asthma medication review.

The results of the psychometric validation of the SRQ presented in this study indicate that Spanish-speaking patients can now also benefit from the RRT and access appropriate support and interventions to reduce SABA overreliance and improve asthma control through adherence to preventer medications.

Discriminant validity was not demonstrated in the current study with regard to patient’s adherence to ICS therapy as measured by the MARS-9 questionnaire and the TAI. However, this concept in particular depends not only on the quality and discriminatory properties of the SRQ itself but also on the psychometric attributes of the questionnaires/measures with which it is compared. As the study cohort consisted of a sample of patients attending for routine consultations, it is possible that the relative stability of this group from the standpoint to their asthma symptoms limited the variability in relation to the variables of interest for the determination of discriminant validity. Additionally, a recent study found that 51.16% of patients with good adherence according to the TAI did not collect at least 80% of their prescribed medication, indicating that the TAI may be overestimating adherence to asthma medication.^15^ On the other hand, discriminant validity as assessed in this study was based on certain hypotheses. Failure to reject the null hypothesis does not imply that the questionnaire does not discriminate; rather, it implies that in our study we were not able to reject the null hypothesis of the assumed relationships between our questionnaire and the MARS-9 and TAI questionnaires. This may be because the hypotheses were not correct or because the sample was not sufficient to reject the null hypothesis.

This study has limitations. The findings are most relevant to Spanish-speaking adults with asthma and cannot be generalized to those speaking other languages. Also, the study population included mainly patients with mild and moderate asthma, with 52.7% of participants not having used their SABA in the past week.

In conclusion, our analyses support the acceptability, reliability, and concurrent validity of the Spanish version of the SRQ with the other validated measures assessing patient perceptions and beliefs about their medication. These results support the use of the Spanish version of the SRQ for the evaluation of Spanish-speaking patients from the standpoint of their potential overreliance on SABA as part of their asthma self-management, enabling early intervention and support.
