# Supplementary material for: Psychometric properties of the Spanish SABA Reliance Questionnaire (SRQ) among patients with asthma
Source: J Allergy Clin Immunol Glob. 2023 Jan 20;2(2):100077. doi: 10.1016/j.jacig.2022.10.008 (PMC10509952; doi:10.1016/j.jacig.2022.10.008)
Supplement: Supplementary Figure 1 [file mmc1.docx]

**Supplementary Figure S1.** Percentage frequency distributions of participants mean scores on the overall SRQ on a 5-point Likert scale.
