# Supplementary material for: Psychometric properties of the Spanish SABA Reliance Questionnaire (SRQ) among patients with asthma
Source: J Allergy Clin Immunol Glob. 2023 Jan 20;2(2):100077. doi: 10.1016/j.jacig.2022.10.008 (PMC10509952; doi:10.1016/j.jacig.2022.10.008)
Supplement: Supplementary Table 1 [file mmc2.docx]

**Supplementary Table S1.** The Spanish SRQ and original items in English^9^.

| Escala tipo Likert de 5 puntos, siendo 1 = totalmente en desacuerdo y 5 = totalmente de acuerdo |
| --- |
| 1. Usar el inhalador de rescate para el asma para tratar los síntomas es la mejor manera de mantener mi asma bajo control. |
| 2. No me preocupo por el asma cuando tengo cerca mi inhalador de rescate para el asma. |
| 3. Mi inhalador de rescate para el asma es el único tratamiento para el asma en el que puedo realmente confiar. |
| 4. Los beneficios de usar mi inhalador de rescate para el asma superan fácilmente cualquier riesgo (efectos no deseados del tratamiento). |
| 5. Prefiero depender de mi inhalador de rescate para el asma que del inhalador de tratamiento diario con corticoides. |

| 5-point Likert-type scale, where 1 = strongly disagree and 5 = strongly agree* |
| --- |
| 1. Using my reliever to treat symptoms is the best way tokeep on top of my asthma |
| 2. I don’t worry about asthma when I have my reliever around |
| 3. My reliever is the only asthma treatment I can really  rely on |
| 4. The benefits of using my reliever inhaler massively outweigh any risks |
| 5. I prefer to rely on my reliever than my preventer inhaler |

  * ©Professor Rob Horne. Please obtain permission from Professor Horne before use.
