# Supplementary material for: Psychometric properties of the Spanish SABA Reliance Questionnaire (SRQ) among patients with asthma
Source: J Allergy Clin Immunol Glob. 2023 Jan 20;2(2):100077. doi: 10.1016/j.jacig.2022.10.008 (PMC10509952; doi:10.1016/j.jacig.2022.10.008)
Supplement: Supplementary Table 2 [file mmc3.docx]

**Supplementary Table S2.** The linguistic adaptation and validation process.^11^

| **Step** | **Process** |
| --- | --- |
| 1 | Preparation: development of document templates and screening for interviewer’s selection |
| 2 | Forward translation: translation of the original RRT from English to Spanish was made by two independent translators |
| 3 | Reconciliation |
| 4 | Back translation: the previously reconciliated Spanish version was independently back-translated to English |
| 5 | Back translation review: the back-translated English version was compared to the original version to ensure the accuracy of the translation and, in case, any discrepancy had been found, adaptations in the reconciliated Spanish version would have been applied |
| 6 | Harmonization: comparison of back translation with the original instrument to highlight discrepancies between the original and its derivative translations, as well as to achieve a consistent approach to translation problems |
| 7 | Cognitive debriefing: the translation was tested for understandability, interpretation, and cultural relevance and alternative wording was explored, in case needed. Thus, cognitive debriefing interviews were conducted in 10 self-reported adult asthma participants, current users of SABA, with a representative range of gender, ages, asthma severity and educational levels |
| 8 | Review of cognitive debriefing results and finalization: analysis and interpretation of the cognitive interview resulted in identifying issues in the Spanish version and amending them |
| 9 | Proofreading: final quality control, to verify that there were no misspells was performed and all formal characteristics of the translated version was verified to be equivalent to the original version |
| 10 | Final report: all the process and results were summarized |
