# Supplementary material for: Psychometric properties of the Spanish SABA Reliance Questionnaire (SRQ) among patients with asthma
Source: J Allergy Clin Immunol Glob. 2023 Jan 20;2(2):100077. doi: 10.1016/j.jacig.2022.10.008 (PMC10509952; doi:10.1016/j.jacig.2022.10.008)
Supplement: Supplementary Table 3 [file mmc4.docx]

**Supplementary Table S3.** Percentage frequency distributions of participant mean scores to the overall SRQ on a 5-point Likert scale (1 being “strongly disagree” and 5 being “strongly agree”)

| **Item and score** | **Distribution, n (%)**  **N=131** | **Mean (SD)** |
| --- | --- | --- |
| 1. Usar el inhalador de rescate para el asma para tratar los síntomas es la mejor manera de mantener mi asma bajo control.   1  2  3  4  5 | 15 (11.45)  21 (16.03)  25 (19.08)  41 (31.30)  29 (22.14) | 3.37 (1.3) |
| 1. No me preocupo por el asma cuando tengo cerca mi inhalador de rescate para el asma   1  2  3  4  5 | 15 (11.45)  22 (16.79)  23 (17.56)  45 (34/35)  26 (19.85) | 3.34 (1.29) |
| 1. Mi inhalador de rescate para el asma es el único tratamiento para el asma en el que puedo realmente confiar   1  2  3  4  5 | 27 (20.61)  27 (20.61)  32 (24.43)  29 (22.14)  16 (12.21) | 2.85 (1.32) |
| 1. Los beneficios de usar mi inhalador de rescate para el asma superan fácilmente cualquier riesgo (efectos no deseados del tratamiento)   1  2  3  4  5 | 11 (8.4)  26 (19.85)  30 (22.90)  53 (40.46)  11 (8.4) | 3.21 (1.11) |
| 1. Prefiero depender de mi inhalador de rescate para el asma que del inhalador de tratamiento diario con corticoides   1  2  3  4  5 | 29 (22.14)  29 (22.14)  18 (13.74)  35 (26.72)  20 (15.27) | 2.91 (1.41) |
| Total SRQ score |  | 15.67 (4.94) |

SD, standard deviation.

Items in the original version:

1. Using my reliever to treat symptoms is the best way tokeep on top of my asthma

2. I don’t worry about asthma when I have my reliever around

3. My reliever is the only asthma treatment I can really rely on

4. The benefits of using my reliever inhaler massively outweigh any risks

5. I prefer to rely on my reliever than my preventer inhaler
